# Prediction of Soil Organic Carbon at the European Scale by Visible and Near InfraRed Reflectance Spectroscopy

**DOI:** 10.1371/journal.pone.0066409

**Published:** 2013-06-19

**Authors:** Antoine Stevens, Marco Nocita, Gergely Tóth, Luca Montanarella, Bas van Wesemael

**Affiliations:** 1 Georges Lemaître Centre for Earth and Climate Research, Earth and Life Institute, UCLouvain, Louvain-la-Neuve, Belgium; 2 SOIL Action, Land Resource Management Unit, Institute for Environment and Sustainability, Joint Research Centre of the European Commission, Ispra, Italy; Lakehead University, Canada

## Abstract

Soil organic carbon is a key soil property related to soil fertility, aggregate stability and the exchange of CO_2_ with the atmosphere. Existing soil maps and inventories can rarely be used to monitor the state and evolution in soil organic carbon content due to their poor spatial resolution, lack of consistency and high updating costs. Visible and Near Infrared diffuse reflectance spectroscopy is an alternative method to provide cheap and high-density soil data. However, there are still some uncertainties on its capacity to produce reliable predictions for areas characterized by large soil diversity. Using a large-scale EU soil survey of about 20,000 samples and covering 23 countries, we assessed the performance of reflectance spectroscopy for the prediction of soil organic carbon content. The best calibrations achieved a root mean square error ranging from 4 to 15 g C kg^−1^ for mineral soils and a root mean square error of 50 g C kg^−1^ for organic soil materials. Model errors are shown to be related to the levels of soil organic carbon and variations in other soil properties such as sand and clay content. Although errors are ∼5 times larger than the reproducibility error of the laboratory method, reflectance spectroscopy provides unbiased predictions of the soil organic carbon content. Such estimates could be used for assessing the mean soil organic carbon content of large geographical entities or countries. This study is a first step towards providing uniform continental-scale spectroscopic estimations of soil organic carbon, meeting an increasing demand for information on the state of the soil that can be used in biogeochemical models and the monitoring of soil degradation.

## Introduction

Human pressure on the soil has now reached the extent to which vital ecosystem services, such as food and fiber production or buffering against increases in greenhouse gas concentrations are at risk [1]–[3]. Soil Organic Carbon (SOC) is recognized as one of the key soil properties reflecting the state of the soil resource [3]. Existing soil maps and inventories are rarely adequate to assess the trends in SOC over time and determine the main driving forces at the scale of a country [4] let alone a continent [5], as the spatial resolution is generally low and many maps are based on outdated and imprecise methods [3]. Hence, high-throughput and cost-effective methods of SOC analysis should be developed to support the implementation of effective soil inventories and production of digital soil maps at the continental scale from which the state of the SOC can be determined in a consistent manner.

Visible and Near InfraRed (Vis-NIR) diffuse reflectance spectroscopy has been applied in soil analysis over the last 20 years [6] and has been demonstrated to accurately measure several soil attributes at minimal costs [7] and with satisfactory analytical errors [8]. Vis-NIR spectroscopy is currently used in laboratory conditions, but its application in-situ and even on air- or space-borne platforms is growing [9]. Vis-NIR reflectance carries information on the organic and inorganic composition of the soil [10] and, due to its integrative nature, has also been proposed as a screening tool for soil quality and fertility diagnosis [11]. Inference is based on multivariate calibration models developed from digital libraries linking Vis-NIR spectral data with reference laboratory measurements [12]. Obviously, these empirical calibrations are only applicable to samples having similar soil composition and spectral characteristics as those in the library and generally and cannot be extrapolated to other soil types [13].

When applying Vis-NIR to assess soil properties in a region of interest, a spectral library representing the local soil diversity needs to be constructed. As a consequence, many local, purpose-specific libraries are being built independently by different research groups using different protocols for soil and spectral analyses. This can produce good results for individual studies, but extrapolation to other areas is difficult. *Mutatis mutandis* the process will have to be repeated over and over again for each study area. This is not efficient and a waste of resources compared to reference methods of soil analyses. This considerably limits the field of applications of local scale spectral libraries so that national and international databases have been or are being developed [14]–[16].

Because soils are extremely variable and the relationship between Vis-NIR spectra and soil attributes can be complex and can vary in space, such databases require very large numbers of samples to be collected to adequately cover soil variation at continental scales [14]. To minimize calibration errors, samples should be analyzed by means of high-standard reference soil analyses and using a standardized spectroscopic measurement protocol [17]. Development costs of such databases can be prohibitive, so that there currently exist only few large scale soil spectral libraries and there are even fewer examples of the use of these libraries as an operational tool for routinely measuring soil properties. However, some initiatives have recently been launched [18], [19]. The world soil spectral library presented in Brown et al. [14] includes 3,794 samples analyzed for SOC content using the Walkley and Black method [20], most of them originating from North America. The ICRAF-ISRIC spectral library contains 4,436 samples from 785 soil profiles distributed across the five continents (only 3,643 samples were analyzed for both chemical properties and soil texture) [16]. SOC was analyzed with the Walkley and Black method. Shepherd and Walsh [12] collected around 1,000 samples for the spectral library of eastern and southern Africa. The Africa Soil Information Service is currently collecting a large number of samples (more than 17,000 so far) from 60 sentinel sites of 100 square km in sub-Saharan Africa that are measured using both soil reference methods and vis-NIR spectroscopy [21]. The Australian library [22] contains 10,677 samples analyzed for total organic carbon using different methods.

In Europe, a large scale soil spectral library has been developed in the framework of the European Land Use/Cover Area frame Statistical Survey (LUCAS) during which ∼20,000 geo-referenced top-soil samples were collected in order to assess the state of the soils across Europe. Thirteen chemical and physical properties, including Vis-NIR reflectance, were analyzed. The database is characterized by a higher sampling density than that of other large scale libraries. All samples were collected following the same sampling protocol and analyzed in a single ISO certified laboratory. SOC content was measured using an automated CN analyzer. To our knowledge, the LUCAS database constitutes to date the most complete and consistent soil spectral library at continental scale. The accuracy of spectroscopic models being limited by *(i)* the number and representativity of the calibration samples, and *(ii)* the quality and consistency of the reference methods, the LUCAS library represents a unique opportunity to evaluate the accuracy of continental-scale soil spectroscopic models. Based on the LUCAS library, we developed the first European-scale calibration models for the prediction of SOC content and analyze model prediction errors.

## Materials and Methods

### Ethics Statement

The LUCAS survey is part of the Community Statistical Programme 2008–2012, based on the decision No 1578/2007/EC of the European Parliament and the Council of the European Union of 11 December 2007 [23]. Data Confidentiality policy is based on the Regulation (EC) No 223/2009 on European statistics (recital 24 and Article 20(4)) of 11 March 2009 [24]. The policy on soil sampling included the clause that upon denial of access, the given point was skipped and a pre-selected alternative location was sampled instead. Field sampling did not involve endangered or protected species.

### The LUCAS SOIL Database

**Figure 1:**
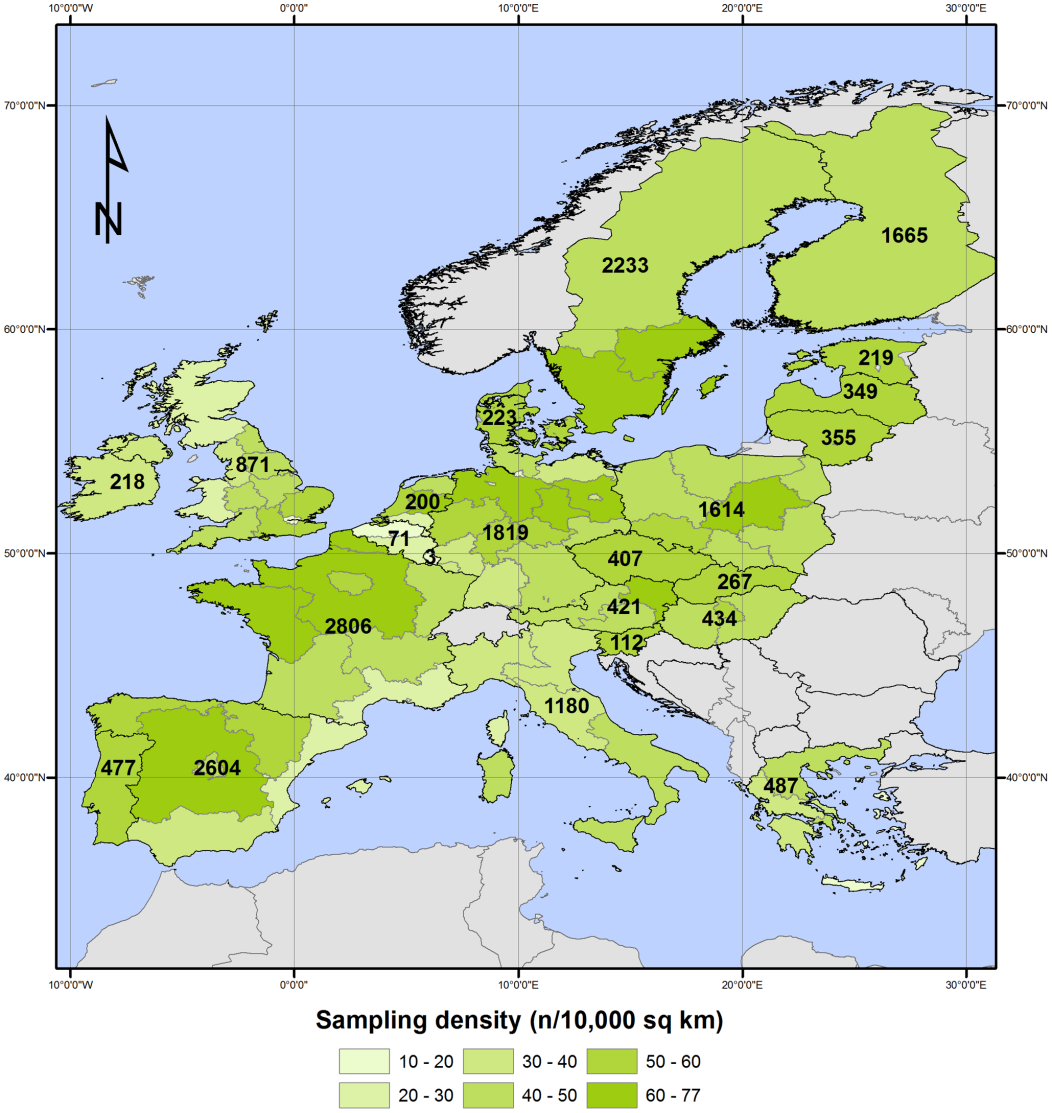
Sampling density of the LUCAS SOIL database per European territorial units, level 1 (NUTS 1). Map labels give the total number of samples per country.

The soil database was compiled as part of the LUCAS survey. Its primary goal was to provide harmonized data on land use/cover in 23 member states of the European Union (EU) by collecting observations in ∼250,000 survey points [25]. About 10% of the points were selected based on environmental variables and accessibility criteria [26] and composite samples consisting of five sub-samples of the top soil (0–30 cm) were taken with a spade following a standardized protocol [26]. Vegetation cover and residues, stones and litter in case of forest land cover were removed before taking a sample. The sampling campaign resulted in the collection of about 20,000 topsoil samples for which geographical coordinates, land use/cover, management and other environmental attributes were retrieved. The mean sample density per European level 1 territorial units (NUTS 1) varies between 11 and 77 samples per 10,000 km^2^, with a mean of 48 samples per 10,000 km^2^ over the 23 EU countries of the survey (Figure 1). While samples are distributed over all land use/cover types, more samples were proportionally taken in cropland soils (Table 1). The surveyed area represents about 68% of the European continent and islands (excluding Russia and Turkey). However, LUCAS samples cover all the major soil types in Europe (Table 2), although Chernozems and Albeluvisols, particularly from Eastern Europe, are underrepresented in the database.

**Table 1:** Number of samples (n), frequency (in% of the total number of samples) and surface (in % of the total surface) occupied by land cover type as defined in the LUCAS survey [31].

Land Cover	n	Frequency (%)	Surface (%)^a^
Artificial	39	<1	5
Bare land	346	2	2
Cropland	8426	44	26
Grassland	4205	22	22
Shrubland	444	2	6
Water areas	12	<1	3
Wetland	90	<1	1
Woodland	5473	29	36

a Percentage of the total surface occupied by land cover type in the 23 EU countries of the LUCAS survey [25].

**Table 2:** Number of samples (n), frequency (in% of the total number of samples) and surface (in % of the total surface) occupied by World Reference Base (WRB) major soil groups [30].

WRB soil type	n	Frequency (%)	Surface (%)^a^	
Unknown	41	<1	–	
Town	23	<1	<1	
Water body	41	<1	<1	
Rock outcrops	3	<1	<1	
Albeluvisols	436	2	7	
Acrisols	45	<1	<1	
Andosols	22	<1	<1	
Arenosols	379	2	1	
Chernozems	193	1	7	
Calcisols	82	<1	<1	
Cambisols	6764	36	25	
Fluvisols	1178	6	5	
Gleysols	502	3	3	
Gypsisols	32	<1	<1	
Histosols	601	3	4	
Kastanozems	0	0	<1	
Leptosols	1078	6	8	
Luvisols	2949	16	11	
Phaeozems	229	1	4	
Planosols	75	<1	<1	
Podzols	3657	19	16	
Regosols	480	3	5	
Solonchaks	55	<1	<1	
Solonetz	19	<1	<1	
Umbrisols	3	<1	<1	
Vertisols	148	1	1	

a Percentage of the total surface occupied WRB major soil groups. Data should be considered approximate: surfaces have been computed using the dominant value of the soil typological units of the European Soil Database [58]. The total land surface considered is the European continent and islands (United Kingdom, Ireland, Iceland, Malta, Sicily, Sardinia, Corsica,…), excluding Russia and Turkey.

### Soil Analyses

All samples were sent to an accredited laboratory (Kecskemét, Hungary) where the following properties were analyzed using ISO standard methods: coarse fragments, particle size distribution, pH in CaCl_2_, pH in water, cation exchange capacity, organic carbon, carbonate, total nitrogen, P, and extractable K content. SOC content (g C kg^−1^) was measured by dry combustion (ISO 10694∶1995) using a vario MAX CN analyzer (Elementar Analysensysteme GmbH, Germany).

### Vis-NIR Measurements and Processing

Absorbance spectra of air-dried and sieved (<2 mm) soil samples were measured with a XDS Rapid Content Analyzer (FOSS NIRSystems Inc., Laurel, MD). The spectrometer is equipped with Si (400–1100 nm) and PbS (1100–2500 nm) detectors, offering 4,200 wavelengths in the Vis-NIR region of the electro-magnetic spectrum. Two scans were acquired and subsequently averaged. For each band, standard deviation between scans was calculated and averaged over the wavelengths. Thirteen spectra with an average standard deviation >0.01 might have been improperly measured and were removed. We corrected the spectra for the shift in absorbance at the splice of the two detectors. The beginning of the Vis (400–500 nm) showed instrumental artifacts and was therefore removed. Several mathematical pre-treatments on the spectra were applied to remove physical variability due to light scattering and enhance features of interest [27]. Firstly, we created two pre-treated spectral matrices by applying Savitzky-Golay (SG) smoothing and first derivative filters [28] with a window size of 101 data points and 3^rd^ order polynomial. Secondly, we applied Standard Normal Variate (SNV) transformation on SG-filtered spectral data [29]. Finally, we kept only one band in twenty (i.e. one every 10 nm), leaving ∼200 predictor variables for model calibration.

### Spectroscopic Models and SOC Predictions

First, samples were separated into mineral and organic soil samples using the FAO definition of organic soil materials [30]. Mineral soil samples were further divided into samples under (*i*) cropland, (*ii*) grassland and (*iii*) woodland land cover. These subsets are based on the land cover classes defined in the LUCAS survey and are consistent with the IPCC/FAO land cover/land use systems [31]. We carried out identical but separate analyses on each of these five subsets (cropland, grassland, woodland, mineral and organic soil samples). We tested several multivariate regression models and spectral pretreatments for predicting the SOC content. Furthermore, we evaluated the potential of (*i*) implementing variable selection procedures through recursive feature elimination *via* random forest and (*ii*) including sand and clay content as auxiliary predictor into the spectroscopic models.

#### Sample selection with the Kennard-Stone algorithm

For each subset, two thirds of the samples were selected for training the spectroscopic models using the Kennard-Stone algorithm [32] and the remaining samples were assigned to the test set for assessing the model’s performance. Based on a spectral distance measure, the Kennard-Stone algorithm selects a set of samples having a uniform distribution over the predictor space and hence that comprise all sources of variation found in the spectral library. The procedure starts by selecting the pair of points that are the farthest apart. They are put in the training set and removed from the list. Then, the remaining points are iteratively assigned to the training set by computing the distance between each unassigned points *i_0_* and training points *i* and finding the point *i_0_* which is the farthest apart from its closest neighbor *i* according to:
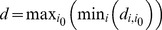
(1)where *d* is a measure of distance. Here, we defined *d* as the Euclidean distance in the normalized score space of the principal components explaining more than 99% of the spectral variation. The principal components were computed on the continuum-removed reflectance spectral matrix [33] to select samples on the basis of their absorption features.

#### Multivariate calibration

Each pre-treated spectral calibration matrix was related to SOC with multivariate regression tools able to deal with high-dimensional and multi-collinear spectral measurements. Using the *caret* package [34] of the R software [35], we ran the following linear and non-linear multivariate models on the training set: partial least square regression, boosted regression tree, random forest, radial-basis support vector machine regression, multivariate adaptive regression splines and Cubist. Details on the latter algorithm, which has shown good prediction accuracy for soil spectral analyses [36], can be found in Quinlan [37]. We used the Cubist GPL C code provided by RuleQuest (RuleQuest Research Pty Ltd, NSW, Australia). The other algorithms are described in Hastie et al. [38] and an overview of their performance for soil spectroscopic inference is given in Viscarra Rossel and Behrens [39]. The models were sequentially developed on a grid of model parameters generated by the *caret* package which provides likely default parameter values. Ten random partitions of the data with a 0.5 selection probability were created and consistently used for leave-one-group-out cross-validation of the models. The best model parameters were determined as the ones producing a model having the smallest value within one standard error of the minimal observed RMSE of cross validation [40].

#### Recursive feature elimination

We tested the potential of a Recursive Feature Elimination (RFE) algorithm based on random forest to select a small set of optimal (and possibly non-collinear) spectral predictors for model calibration. The RFE procedure, as implemented in the *caret* package, performs a backward selection of the variables by ranking their importance to an initial model run using all the predictors [41]. The algorithm builds several calibration models that use the *p_i_* most important predictors, where *p_i_* is an element of a predetermined sequence *{p_1_,p_2_,…,p_n_}* of possible numbers of predictors. The set of predictors *p*_i_ producing the best model amongst the candidate models is retained.

#### Auxiliary predictor

We assessed the possibility to improve the models by adding another predictor to the spectral matrix, which to be useful should be readily available [14]. We tested sand and clay content as auxiliary predictors. Particle size fractions are unlikely to change much over time at the sample location and hence could directly be exploited in models predicting the SOC content of samples collected during a future resampling of the LUCAS database. In order not to overweigh in the multivariate model the spectral data compared to the auxiliary predictor, we computed the principal component scores of the spectral matrix, retained the scores explaining more than 99% of the variation, attached the auxiliary predictor to the scores and scaled the resulting matrix [42]. When RFE is applied, we assumed that the spectral matrix has been reduced to its intrinsic dimensionality so that the predictor matrix was only scaled, without performing the principal component step. This approach was tested only for mineral soils since texture analyses were not realized for organic soil samples.

#### Assessing model performance

Multivariate models were validated with the test set and their quality assessed by means of the following statistics [43]:
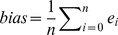
(2)

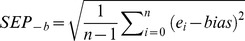
(3)

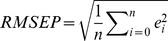
(4)

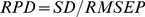
(5)where *e*_i_ is the residuals (i.e. prediction error) of sample *i* in the test set, *n* is the number of observations, *SD* is the standard deviation of the observations. The *SEP_-b_* (Eq. 3) is the standard error of prediction corrected for bias (i.e. the difference between predicted and observed means, Eq. 2) and is equivalent to the standard deviation of the predicted residuals. The *SEP_-b_* and bias represent two independent components of the Root Mean Square of Prediction (*RMSEP*, Eq. 4). The Ratio of Performance to Deviation (*RPD*, Eq. 5) is a way of normalizing *RMSEP*’s to compare calibration models where the measured variables have different ranges or variances. We used the *RMSEP* (Eq. 4) to rank all pre-treatments and multivariate calibration models and choose the best modeling approach.

#### Reproducibility of the reference and spectral methods

All soil analyses were replicated once for 25 randomly-selected samples which allowed estimating the reproducibility (or intermediate precision) of the reference and spectral analyses methods (i.e. repeatability+between-runs error) using [44]:
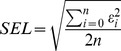
(6)where *SEL* is the Standard Error of Laboratory and ε_i_ is the difference between duplicate measurements/predictions of sample *i*.

## Results and Discussion

### Exploratory Analysis of the LUCAS Database

Mineral samples have a mean SOC content of 29 g C kg^−1^, a median at 19.6 g C kg^−1^ and a highly skewed SOC distribution with 75% of the samples below 35 g C kg^−1^ (Table 3). The SOC values of mineral samples of the LUCAS library are relatively higher than in other large scale spectral library, mainly because many samples were collected in organic-rich soils of northern Europe (Figure 1). The African [12], Australian [22] and world [14] spectral libraries have a median SOC content of respectively 12, 6 and 4.7 g C kg^−1^. SOC content of organic samples in the database ranges from 156 to 587 g C kg^−1^ with a mean of 387 g C kg^−1^.

**Table 3:** Summary statistics of soil properties available in the LUCAS database, for mineral and organic soil materials.

Property	Unit	Mean	SD^a^	Min	Q25^b^	Q50^c^	Q75^d^	Max	Skew	ρ_PC1_ e	ρ_PC2_ e	ρ_PC3_ e	n^f^
		**Mineral soils**											
SOC	g kg^−1^	29.4	28.9	0.0	12.3	19.6	34.7	199.2	2.67	0.08	−0.55	0.16	17937
N	g kg^−1^	2.2	1.6	0.0	1.2	1.7	2.6	16.2	2.44	−0.01	−0.44	0.17	17937
clay	g kg^−1^	18.9	13.0	0.0	8.0	17.0	27.0	79.0	0.91	−0.45	0.23	−0.03	17937
silt	g kg^−1^	38.2	18.3	0.0	25.0	37.0	51.0	92.0	0.21	0.07	0.27	−0.04	17937
sand	g kg^−1^	42.9	26.1	1.0	19.0	42.0	64.0	99.0	0.19	0.17	−0.30	0.04	17937
CaCO_3_	g kg^−1^	54.6	128.4	0.0	0.0	1.0	16.0	944.0	2.87	−0.32	0.12	0.05	17937
pHw	–	6.3	1.3	3.4	5.2	6.3	7.5	10.1	−0.13	−0.50	0.27	−0.07	17937
CEC	cmol^+^kg^−1^	14.1	10.5	0.0	6.8	11.7	18.7	137.0	1.94	−0.50	−0.08	0.13	17937
		**Organic soils**											
SOC	g kg^−1^	387.1	101.2	156.4	297.3	401.1	475.0	586.8	−0.25	−0.46	0.35	0.26	1099
N	g kg^−1^	15.5	5.7	3.1	11.2	14.5	19.0	38.6	0.72	−0.53	−0.10	−0.05	1099
CaCO_3_	g kg^−1^	2.9	19.9	0.0	0.0	0.0	1.0	418.0	14.63	0.03	0.02	−0.11	1099
pHw	–	4.5	0.7	3.2	4.0	4.3	4.7	7.5	1.35	−0.23	−0.09	−0.54	1099
CEC	cmol^+^kg−^1^	42.0	33.0	0.0	23.8	31.8	42.5	234.0	2.54	−0.39	0.03	−0.48	1099

a Standard Deviation;

b lower quartile;

c median;

d upper quartile,

e correlation of PC1-3 scores with the soil properties;

f number of samples.

**Figure 2:**
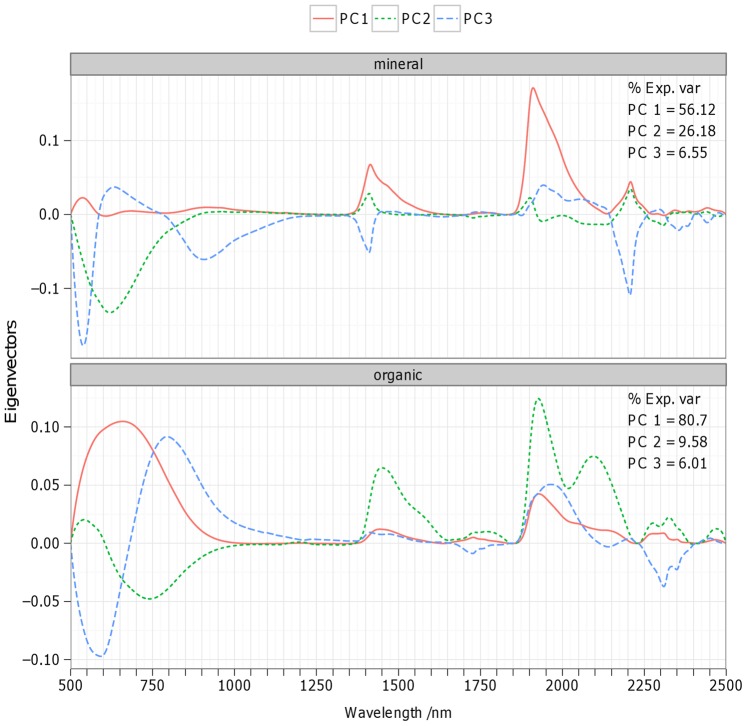
Eigenvectors and eigenvalues of the first three principal components of continuum-removed spectra. The principal component analysis has been realized separately for mineral (top panel) and organic (bottom panel) soil materials.

To analyze the spectral variation included in the database, we performed a principal component analysis on the continuum-removed reflectance of mineral and organic samples [33]. The eigenvectors of the three first principal components (PC) show diagnostic variations across the Vis-NIR spectrum that can be linked to soil properties (Figure 2). For mineral soils, the first PC, explaining 56% of the spectral variation, shows important peaks that are associated to overtones of O-H and H-O-H stretch vibrations of free water (1455 and 1915 nm) and overtones and combinations of O-H stretch and metal-OH bends in the clay lattice (1415 and 2207 nm), which express spectral differences between illite and smectite clay minerals [39]. Hence, the scores of PC1 are strongly correlated with soil properties related to clay mineralogy such as clay content (ρ = −0.45), cation exchange capacity and pH in water (ρ = −0.5; Table 3). The eigenvector of PC2 (explaining 26% of the spectral variation) is dominated by one prominent feature centered at 620 nm that can be attributed to various organic compounds [10], explaining the negative correlation of PC2 scores with OC (ρ = −0.55) and N (ρ = −0.44; Table 3). While the scores of PC3 show only weak correlations with the measured soil properties (Table 3), the third PC can be related to the absence/presence of iron oxides as indicated by well-defined peaks in the eigenvector of PC3 around 540, 640 and 900 nm (Figure 2) caused by variations in the shape of the absorptions due to electronic transitions of goethite iron oxide at 620 and 920 nm [39].

For organic soils, the first PC scores are strongly correlated with OC (ρ = −0.46) while PC2-3 scores show weaker correlations with OC (ρ = 0.26–0.35; Table 3). The eigenvectors of PC1-3 display oscillations between 500 and 900 nm that are related to variations in the size and width of the organic matter absorption feature in the visible region (Figure 2). Other important features in the eigenvectors can be distinguished at 1450 nm, 1760 nm and between 1900 and 2500 nm corresponding to vibrations of C-O, O-H, C = O,C-H and N-H bonds that are present in organic compounds such as proteins, starch, cellulose, humic acids and lignin [45]. These absorptions are however difficult to attribute to a single component since they are greatly overlapping in the NIR. Since these organic soils have generally very low clay content, there is no visible feature at 2207 nm due to O-H and metal-O-H bonds in clay mineral lattices. The eigenvector of PC2 shows three local minima around 1450, 2100 and 2300 nm that can be assigned to lignin and cellulose [46] and may therefore account for spectral variation related to the decomposition stage of organic matter (arising e.g. from difference between forest and wetland samples). This preliminary analysis of European-scale spectral variation demonstrates that soil spectra are tightly linked with key soil properties, which supports thereby the development of spectral prediction models.

### Multivariate Models for SOC Prediction

We compare here the prediction ability of the different models and pre-treatments tested for the subsets. For cropland, mineral and organic soils, the lowest prediction errors were achieved by models using the first derivative of the spectral matrix, while for grassland and woodland soil samples, the best models used the primary absorbance spectra (Figure S1). Overall, SNV transformation did not noticeably improve the accuracy of the models (Figure S1). Model performance varied greatly with the predictors included (Figure S2). Using sand content in addition to the spectral matrix improved grassland and woodland models compared to models using spectral data only, with a median decrease in *RMSEP* of ∼1 g C kg^−1^ for grassland soils and ∼4 g C kg^−1^ for woodland soil, while no clear improvement could be observed for cropland and mineral soil models (Figure S2). Using clay content and the spectral matrix in the models allowed to decrease *RMSEP* of grassland soil predictions with a median of ∼1 g C kg^−1^ but no improvement was observed for other subsets (Figure S2).

Recursive feature elimination provided no overall increase in prediction accuracy for models using spectral data only. This is to be expected since most of the multivariate models that we tested (boosted regression tree, Cubist, multivariate adaptive regression splines) include an internal feature selection method. However, models using RFE in combination with sand and clay content showed clear improvements in accuracy compared to models using the spectral matrix only and the spectral matrix in combination with particle size fractions (Figure S2). This is probably related to the fact that RFE, by reducing the dimensionality of the spectral matrix and by keeping the relevant information for SOC prediction, allowed increasing the relative weight of the auxiliary predictor in the models compared to the spectral matrix. Cubist, closely followed by support vector machine regressions, produced the most accurate predictions (i.e. have lower *RMSEP*) for grassland and woodlands soils, while Cubist regressions performed slightly less for cropland and mineral subsets (Figure S3). This confirms the good performance of support vector machine regression and Cubist in predicting soil properties compared to other multivariate calibration models [36], [39]. For organic soils, Cubist and partial least square regression showed the best prediction abilities (Figure S3).

### Performance of the Best Spectroscopic Models

Prediction performance statistics of the best models (i.e. having the lowest *RMSEP*’s) with and without auxiliary predictors are given in Table 4. The lowest *RMSEP*’s (Eq. 4) were obtained for cropland soils (4–4.9 g C kg^−1^), followed by grassland (6.4–9.3 g C kg^−1^), mineral (7.3–8.9 g C kg^−1^), woodland (10.3–15 g C kg^−1^) and organic soils (50.6 g C kg^−1^; Table 4). The difference in *RMSEP* between the subsets reflected the dependence of the model errors on (*i*) calibration size and (*ii*) the variance of observed SOC values. Hence, cropland, grassland and mineral soils, characterized by a large number of samples and small variance were better predicted than woodland and organic soils (Table 4). The accuracy of spectroscopic models increased with the number of calibration sample [12], [47] because a large sample size allows to better describe the soil complexity of a given area. The tendency of *RMSEP* to increase with SOC variance as observed in the LUCAS database is also well documented [8]. Datasets characterized by larger SOC variances usually cover larger areas or areas with an important variation in soil properties, which may be detrimental to SOC prediction models. However, since all subsets cover the same geographical extent, it is more probable that SOC variation itself and SOC concentration rather than soil diversity explain the increase in *RMSEP* from cropland to organic soils [8], [48], [49].

**Table 4:** Performance of the best spectroscopic models as measured against the test set.

Subset	Treatment^a^	MVC^b^	Predictor^c^	SD^d^	RMSEP^e^	Bias^f^	SEP_-b_ g		RPD^h^	R^2^	N^i^
Cropland	SG1	svm	spc	8.6	4.9	0.2	4.9	1.74		0.67	2828
Cropland	SG1+SNV	svm	rfe+clay	8.6	4.0	0.1	4.0	2.17		0.79	2828
Grassland	SG1	svm	spc	17.4	9.3	−0.9	9.3	1.86		0.71	1383
Grassland	SG0	cubist	rfe+sand	17.4	6.4	0.1	6.4	2.70		0.87	1383
Woodland	SG1	svm	spc	29.8	15.0	0.8	15.0	1.99		0.75	1564
Woodland	SG0	cubist	rfe+sand	29.8	10.3	1.1	10.3	2.88		0.89	1564
Mineral	SG1	svm	spc	19.1	8.9	0.2	8.9	2.13		0.78	6053
Mineral	SG1	svm	rfe+sand	19.1	7.3	0.1	7.3	2.62		0.86	6053
Organic	SG1+SNV	cubist	spc	100.8	50.6	−10.9	49.5	1.99		0.76	368

a Spectral transformation (SG0 =  Savitzky-Golay smoothing; SG1 =  Savitzky-Golay first derivative; SNV = standard normal variate);

b Multivariate Calibration Model (svm = support vector machine regression; cubist = Cubist);

c Predictor used in the models (spc = spectral matrix; rfe = spectral matrix with bands selected by recursive feature elimination);

d Standard Deviation of the observations (g C kg^−1^);

e Root Mean Square Error of Prediction (g C kg^−1^; Eq. 4);

f Bias (g C kg^−1^; Eq. 2);

g Standard Error of Prediction (g C kg^−1^; Eq. 3);

h Ratio of Performance to Deviation (Eq. 5);

i Number of validation samples.

**Figure 3:**
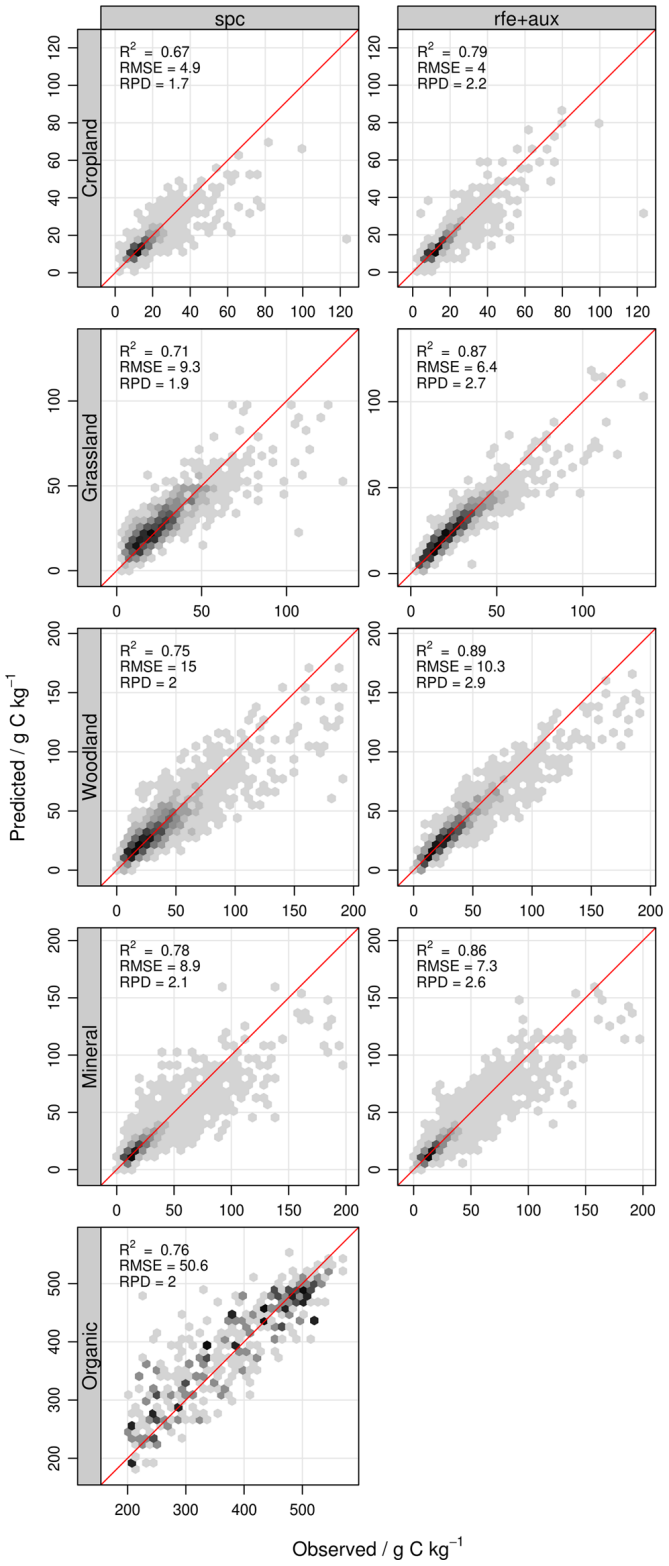
Predicted SOC content as a function of observed SOC content in test sets. Model predictions are shown for models with (rfe+aux, right panels) and without auxiliary predictors (spc, left panels).

The bias (Eq. 2) of the spectroscopic models was very low in absolute value (<1.1 g C kg^−1^ for mineral soils, Table 4) compared to the standard error of prediction corrected for bias (*SEP_−b_*; Eq. 3), indicating that a large portion of the error was due to the residual variance. Residuals tend to increase with increasing SOC content, except for organic soils (Figure 3). While such increase in model residuals could be attributed to an increase in analytical error with SOC content [12], we rather suggest that this was caused by the skewed distribution of SOC content of mineral soils (Table 3) because predictions at high SOC content were affected by a strong bias (Figure 3). This often occurs when predicted samples are under-represented in the training set [50]. Organic soil samples, having only a small negative skewness (Table 3), did not show an increase in prediction residuals with SOC content.

The accuracy of the models developed from the LUCAS library compared very well to other published results with Ratio of Performance to Deviation (*RPD*; Eq. 5) ranging from 1.74 to 2.88 and R^2^ from 0.67 to 0.89 (Table 3). SOC spectroscopic models reported in the literature achieve an R^2^ between predicted and observed values ranging from 0.66 to 0.96 (average of 0.79) and *RPD* values ranging from 1.44 to 4.2 [51], [52]. Brown et al. [14] obtained a *RMSEP* of 7.9–9 g C kg^−1^ for global SOC spectroscopic models of mainly mineral soils. The accuracy of spectroscopic models is usually negatively related to the heterogeneity of the samples in a given spectral library [8], [12]. This relation explains the relatively low accuracy achieved by large-scale calibrations compared to the one that can be potentially obtained by local-scale (i.e. field, landscape-scale) spectroscopic models. The main reason for the poor performance on heterogeneous soils is that absorption features associated to organic matter can be altered and/or masked by other components of the soil (e.g. iron oxides, clay mineralogy) or can change with the chemical composition or quality of the organic matter [50]. Hence, for heterogeneous soils there is no univocal relationship between SOC content and soil spectra.

**Figure 4:**
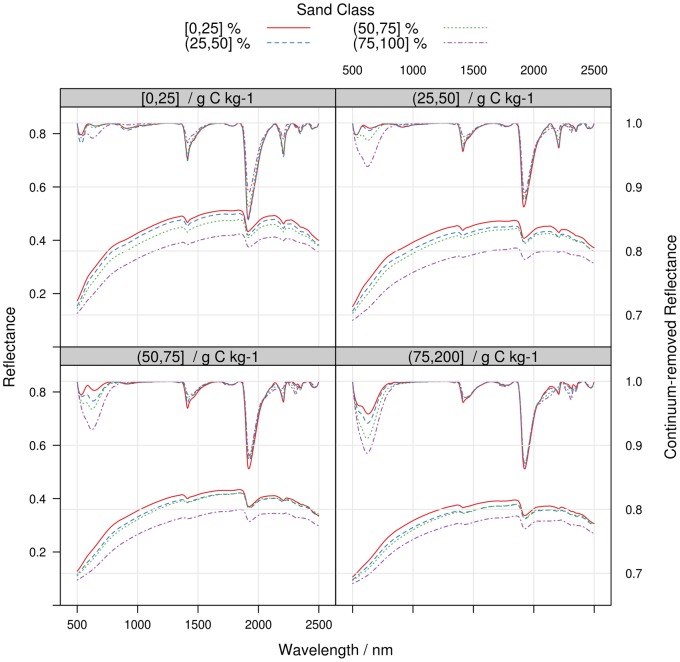
Mean reflectance (left scale) and continuum-removed reflectance (right scale) spectra of LUCAS mineral soil samples, computed for arbitrary sand and SOC classes. The sand classes are 0–25%, 25–50%, 50–75%, 75–100% and the SOC classes are 0–25 g C kg^−1^, 25–50 g C kg^−1^, 50–75 g C kg^−1^, 75–200 g C kg^−1^. Each panel regroups samples of a given SOC interval.

**Figure 5:**
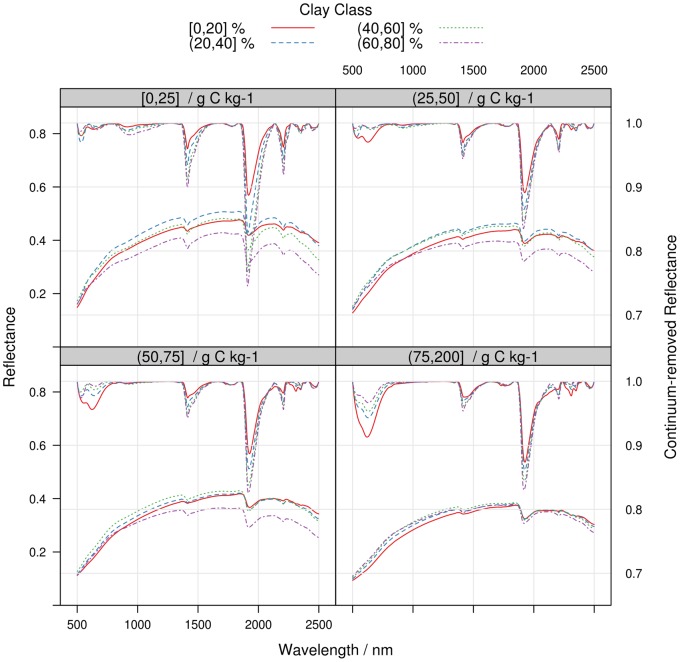
Mean reflectance (left scale) and continuum-removed reflectance (right scale) spectra of LUCAS mineral soil samples, computed for arbitrary clay and SOC classes. The clay classes are 0–20%, 20–40%, 40–60%, 60–80% and the SOC classes are 0–25 g C kg^−1^, 25–50 g C kg^−1^, 50–75 g C kg^−1^, 75–200 g C kg^−1^. Each panel regroups samples of a given SOC interval.

To better understand how the spectral response of SOC can be affected by variations in other soil properties, we computed the mean reflectance and continuum-removed reflectance of mineral samples grouped by classes of SOC, sand and clay content (Figure 4–5). Mean reflectance values tend to decrease with both sand and SOC content, so that variation in the spectra that are due to sand content can be confounded with spectral variations due to an increase in SOC content (Figure 4). Sand is a featureless property. However, an increase in sand content typically increases light scattering, which in turn diminishes spectral baseline height and enhances weak absorptions through an increased path length [53]. This effect can be clearly observed in continuum-removed reflectance values (Figure 4): for the same amount of SOC, the absorption feature between 500 and 800 nm that is linked to SOC content is enhanced as sand content increases. Similarly, variations in clay content induce large differences in spectral shape for the same class of SOC content (Figure 5). In each SOC class, one can indeed observe an increase in the depth of absorptions related to O–H and metal–OH in the mineral crystal lattice and O-H in water (1415, 1455, 1915 and 2207 nm) with the increase in clay content. Conversely, the SOC absorption between 500 and 800 nm is progressively masked as the clay content increases. Generally, the albedo of the mean spectra tends to increase with clay content until 60% of clay and decreases thereafter. Differences in albedo are more pronounced for samples with SOC contents below 50 g C kg^−1^ (Figure 5).

**Figure 6:**
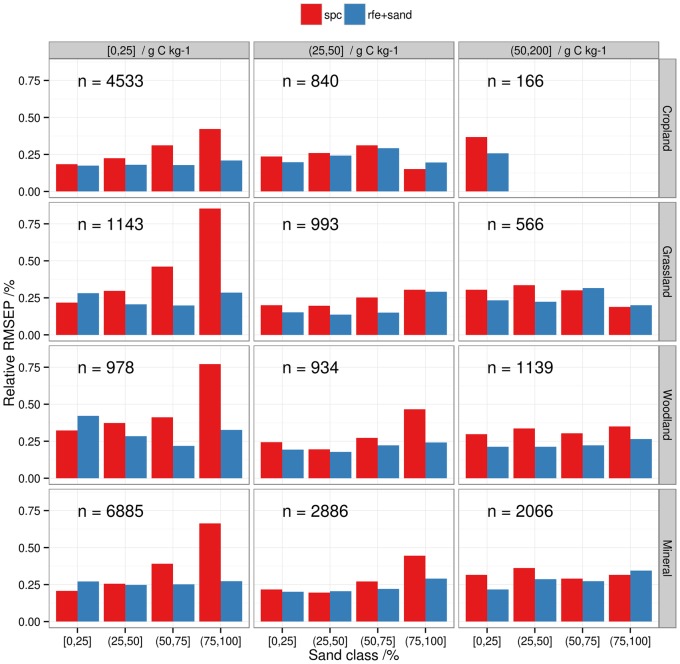
Relative Root Mean Square Error of Prediction (*RMSEP*) per land cover, for arbitrary classes of SOC and sand content. The sand classes are 0–25%, 25–50%, 50–75%, 75–100% and the SOC classes are 0–25 g C kg^−1^, 25–50 g C kg^−1^, 50–200 g C kg^−1^. The relative *RMSEP* is the *RMSEP* divided by the mean of observed SOC values of models developed with (red bars) and without auxiliary predictors (blue bars). Each panel regroups mineral samples of a given SOC interval and land cover type. The number of training samples (n) for each class of SOC content is given in each panel.

The differences in spectral response observed in Figure 4–5 had logically a strong impact on model errors. To illustrate this, we computed the relative *RMSEP* for mineral soil models for intervals of SOC and sand content. The relative *RMSEP* is the *RMSEP* divided by the mean of the observed SOC content in a given class. For models using the spectra only for prediction, the relative *RMSEP* of the models was stable across the SOC content classes but it increased with the sand content (Figure 6). This confirms the results of other studies [48], [54] that found larger SOC prediction errors for soils with the highest sand contents. The effect of sand content on SOC prediction accuracy was more pronounced at low SOC content due to the relatively low absorption rates of organic matter and the masking from other soil components ([55]; Figure 4–5). It is therefore expected that spectral libraries of soils characterized by a low SOC content will perform poorly when samples have large variations in particle size distribution. It can be also observed that the use of sand content as auxiliary predictor drastically improved model predictions for sandy soils (Figure 6), explaining the increase in model accuracies compared to models based on the spectral matrix only (Table 4).

### Reproducibility Error

We assessed the reproducibility error of the models with a set of duplicate samples. The error of reproducibility (*SEL*, Eq. 6) of the reference method was estimated at 1.5 g C kg^−1^ for cropland, 0.8 g C kg^−1^ for grassland, 2.9 g C kg^−1^ for woodland, 2 g C kg^−1^ for mineral and 11.6 g C kg^−1^ for organic soils (Table 5). The reproducibility of SOC predictions by the spectroscopic models was roughly similar, with values ranging from 1.2 to 9.1 g C kg^−1^ (Table 5). These relatively low values suggested that spectroscopic models are robust through time and only a small component of the error budget can be attributed to measuring conditions (e.g. temperature and humidity in the laboratory). Both analytical techniques showed an increase in reproducibility error with the SOC content.

**Table 5:** Reproducibility of SOC estimates (g C kg^−1^; Eq. 6) of the reference method and the spectroscopic models with (rfe+aux) and without (spc) the use of auxiliary predictors.

Subset	Reference	spc	rfe+aux	n^a^
Cropland	1.5	1.8	0.9	13
Grassland	0.8	1.4	1.7	5
Woodland	2.9	2.1	2.4	4
Mineral	2	1.8	2.4	22
Organic	11.5	9.1	–	3

a Number of duplicate samples.

### Implication for SOC Spectroscopic Assessment at Large Scale

The prediction error of the spectroscopic models was ∼5 times larger than the reproducibility error of the reference method (Table 3–4) while ratio of *RMSEP* to *SEL* of 1 to 3.5 have been reported in local studies [51]. This clearly indicates that it is currently difficult to produce spectroscopic models of SOC content that are valid for large areas while sufficiently accurate to be useful for most applications at fine scales. However, the models proved to have low biases (Table 4) and hence could be used to estimate the mean SOC content of large areas since the variance of the model residuals is reduced by averaging [6].

Increasing the number of training samples improves the prediction accuracy [12] because a higher number of samples will better describe the soil variability in a given area. Despite the relatively high sampling density of the LUCAS database (Figure 1; Table 1–2), large prediction errors are still observed (Table 4) and it is unlikely that including more samples in the database will significantly improve the prediction for the geographical entities covered by the current LUCAS survey. The two main reasons for this are that: *(i)* soils are complex materials with a strong spatial structure and, as stated above, the relationship linking soil properties with soil spectra is not stationary, so that large-scale spectroscopic models cannot achieve the same level of accuracy as for more homogeneous materials like plants or other agricultural commodities [13] and *(ii)* the natural positive skew of SOC values induces a large model bias at high SOC content. Rather than increasing the number of samples in soil spectral libraries, further efforts should be deployed towards the development of calibration models that are capable of identifying local patterns of spectral variation in large scale libraries because developing a universal calibration model for SOC prediction is unlikely. We showed that a promising approach consists in including readily available soil covariates in the spectroscopic modeling, although other strategies should be explored, such as simple subsetting of samples by soil type or SOC content [49], [56]. Ideally, covariates should be linked with important spectrally-active soil components (e.g. mineralogy, texture, iron content) or with pedogenetic factors such as climate and land cover.

### Conclusion

The LUCAS soil database is the result of a harmonized soil survey conducted on a relatively dense sampling grid over 23 member states of the European Union. This database represents currently the most comprehensive soil spectral library at continental scale using a uniform protocol for both chemical and spectral analyses. Still, SOC spectroscopic models showed relatively large errors (>4 g C kg^−1^) compared to established methods of SOC analysis, suggesting that accurate SOC predictions based on large scale spectral libraries will be hard to achieve. Prediction errors were found to be related to SOC variation, SOC distribution (skewness) and variation in other soil properties such as sand and clay content. These findings strongly suggest that vis-NIR spectral data alone do not contain enough information to get accurate predictions of soil properties at large scales. Hence, to develop spectroscopy as a valuable tool for soil analyses, further research should be directed towards the development of strategies that can address this issue, such as the use of additional predictors in the modeling.

Despite these difficulties, large spectral libraries can be very valuable to (*i*) build local and more accurate spectroscopic models that are specific to a given geographical entity or soil type and (*ii*) develop spectroscopic models able to quickly produce SOC predictions for estimate accurately SOC means across regions or countries, due to the unbiasedness of the method. The LUCAS spectral library will be made publicly available for non-commercial purpose through the European Soil Data Centre (http://eusoils.jrc.ec.europa.eu/projects/lucas/data.html).

Although not addressed in this paper, there are indications that other key soil properties identified by the Global Soil Map community such as clay content, pH and cation-exchange capacity [3] can simultaneously be analyzed using Vis-NIR spectroscopy [8]. As similar large scale spectral libraries are being developed in USA [57], Africa [16], and Australia [22], spectral analyses will provide consistent soil measurements for a large portion of soils across the globe. However, in order to develop soil spectroscopy into an operational tool producing harmonized data across laboratories and environments, we urgently need consultation and cooperation to define internationally agreed standards for soil spectral analyses, including norms for instrumentation, sampling preparation, reference materials, measuring set-up, quality checks and calibration transfer methods.

## Supporting Information

Figure S1 **Box-and-whisker plots of the Root Mean Square Error of Prediction (** ***RMSEP*** **)** **as a function of the pretreatments.** Each panel presents separately the results obtained for cropland, grassland, woodland, mineral and organic models. Pretreatments: SG0 =  Savitzky-Golay smoothed absorbance; SG1 =  Savitzky-Golay first derivative; A = absorbance; SNV = Standard Normal Variate.
(TIF)

Figure S2 **Box-and-whisker plots of the Root Mean Square Error of Prediction (** ***RMSEP*** **) as a function of the predictors.** Each panel presents separately the results obtained for cropland, grassland, woodland, mineral and organic models. Predictors: spc = spectral matrix; rfe = spectral matrix with bands selected by recursive feature elimination.
(TIF)

Figure S3 **Box-and-whisker plots of the Root Mean Square Error of Prediction (** ***RMSEP*** **) as a function of the multivariate calibration approach.** Each panel presents separately the results obtained for cropland, grassland, woodland, mineral and organic models. Multivariate models: pls = partial least square regression; cubist = Cubist; mars = multivariate adaptive regression splines; brt = boosted regression tree; rf = random forest; svm = support vector machine.
(TIF)
