# Supplementary figures and images for: Prediction of Soil Organic Carbon at the European Scale by Visible and Near InfraRed Reflectance Spectroscopy
Source: PLoS One. 2013 Jun 19;8(6):e66409. doi: 10.1371/journal.pone.0066409 (PMC3686688; doi:10.1371/journal.pone.0066409)

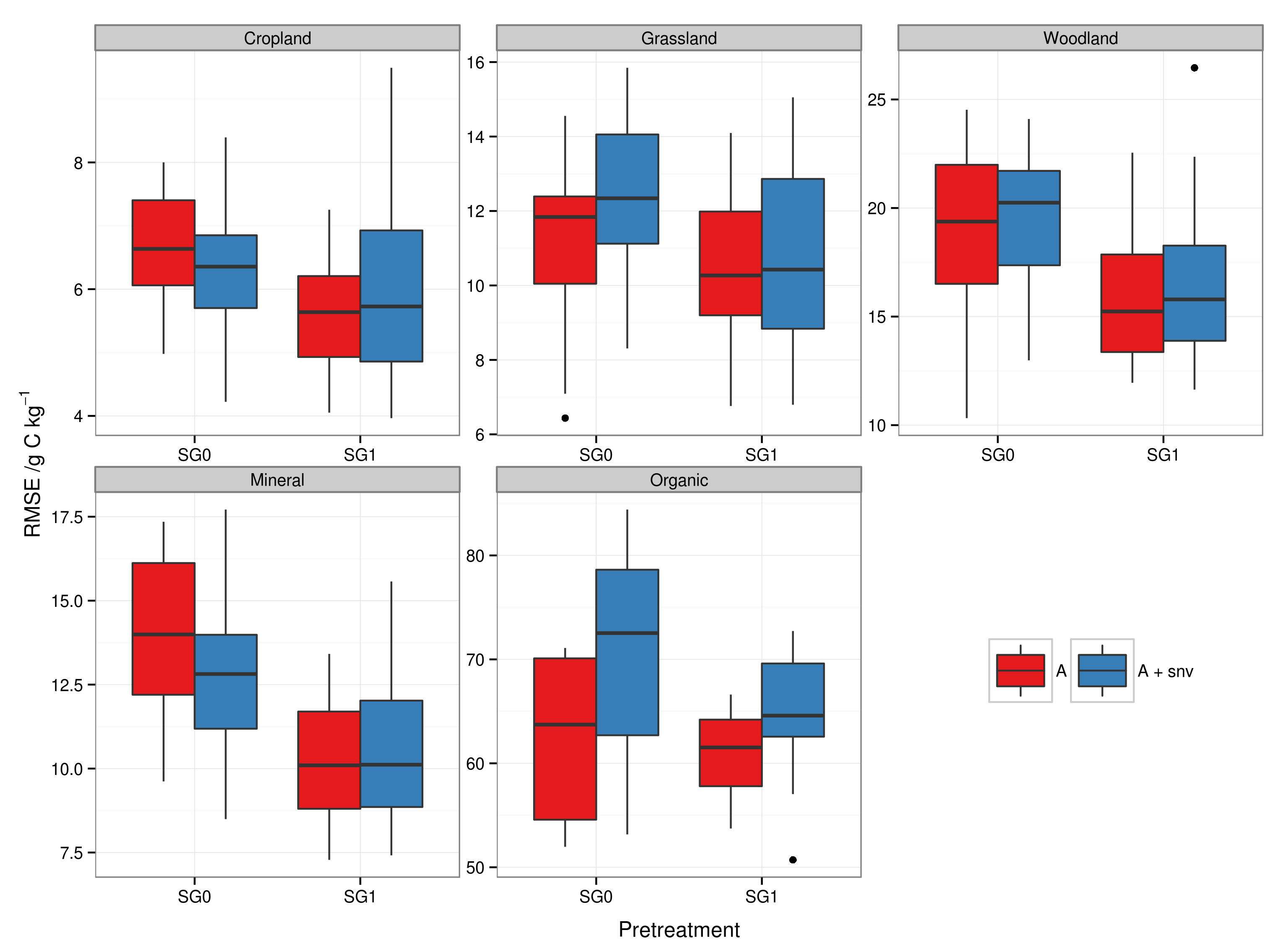

Supplement: Figure S1 — Box-and-whisker plots of the Root Mean Square Error of Prediction ( RMSEP ) as a function of the pretreatments. Each panel presents separately the results obtained for cropland, grassland, woodland, mineral and organic models. Pretreatments: SG0 = Savitzky-Golay smoothed absorbance; SG1 = Savitzky-Golay first derivative; A = absorbance; SNV = Standard Normal Variate. (TIF) [file pone.0066409.s001.tif]

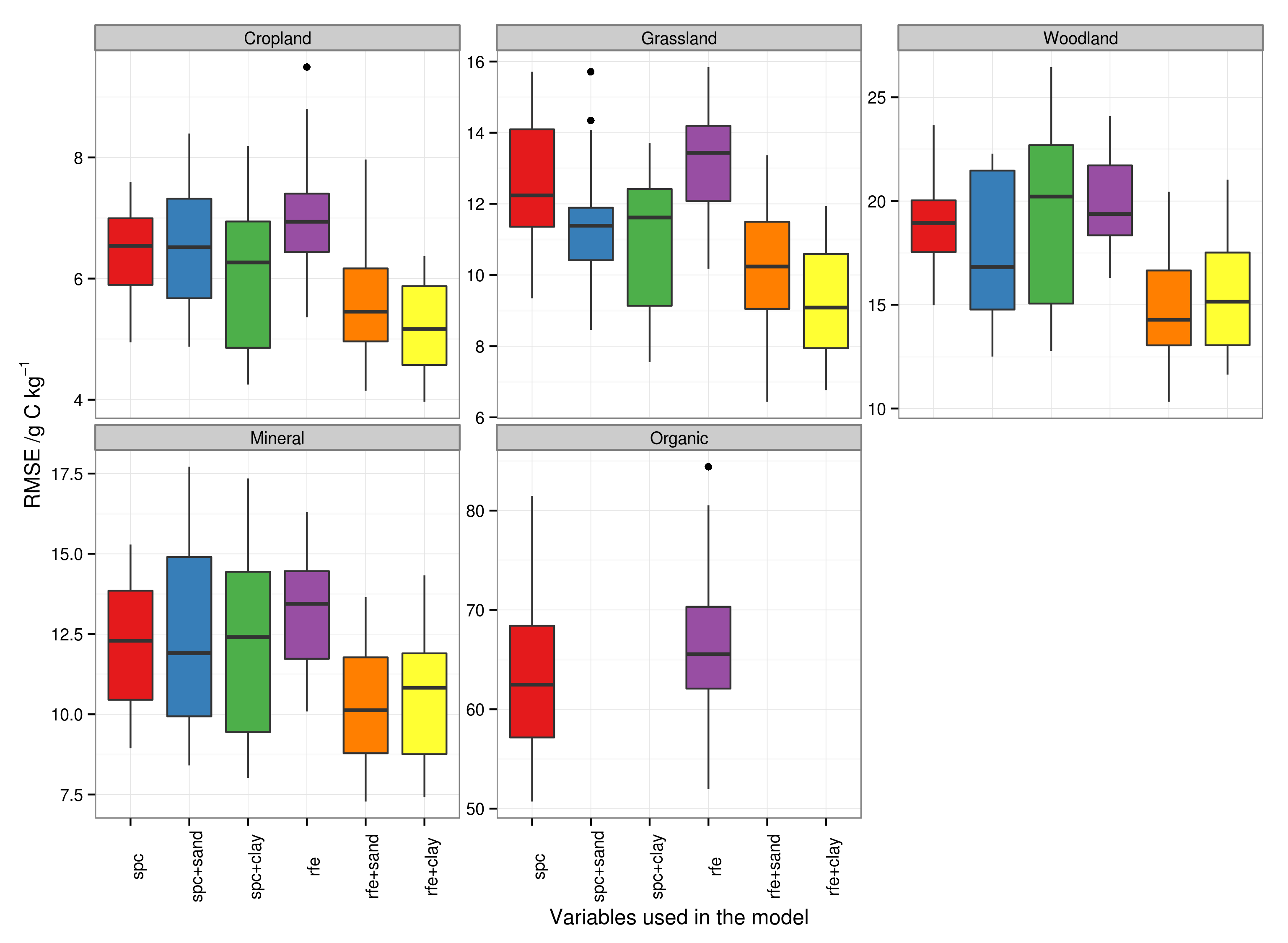

Supplement: Figure S2 — Box-and-whisker plots of the Root Mean Square Error of Prediction ( RMSEP ) as a function of the predictors. Each panel presents separately the results obtained for cropland, grassland, woodland, mineral and organic models. Predictors: spc = spectral matrix; rfe = spectral matrix with bands selected by recursive feature elimination. (TIF) [file pone.0066409.s002.tif]

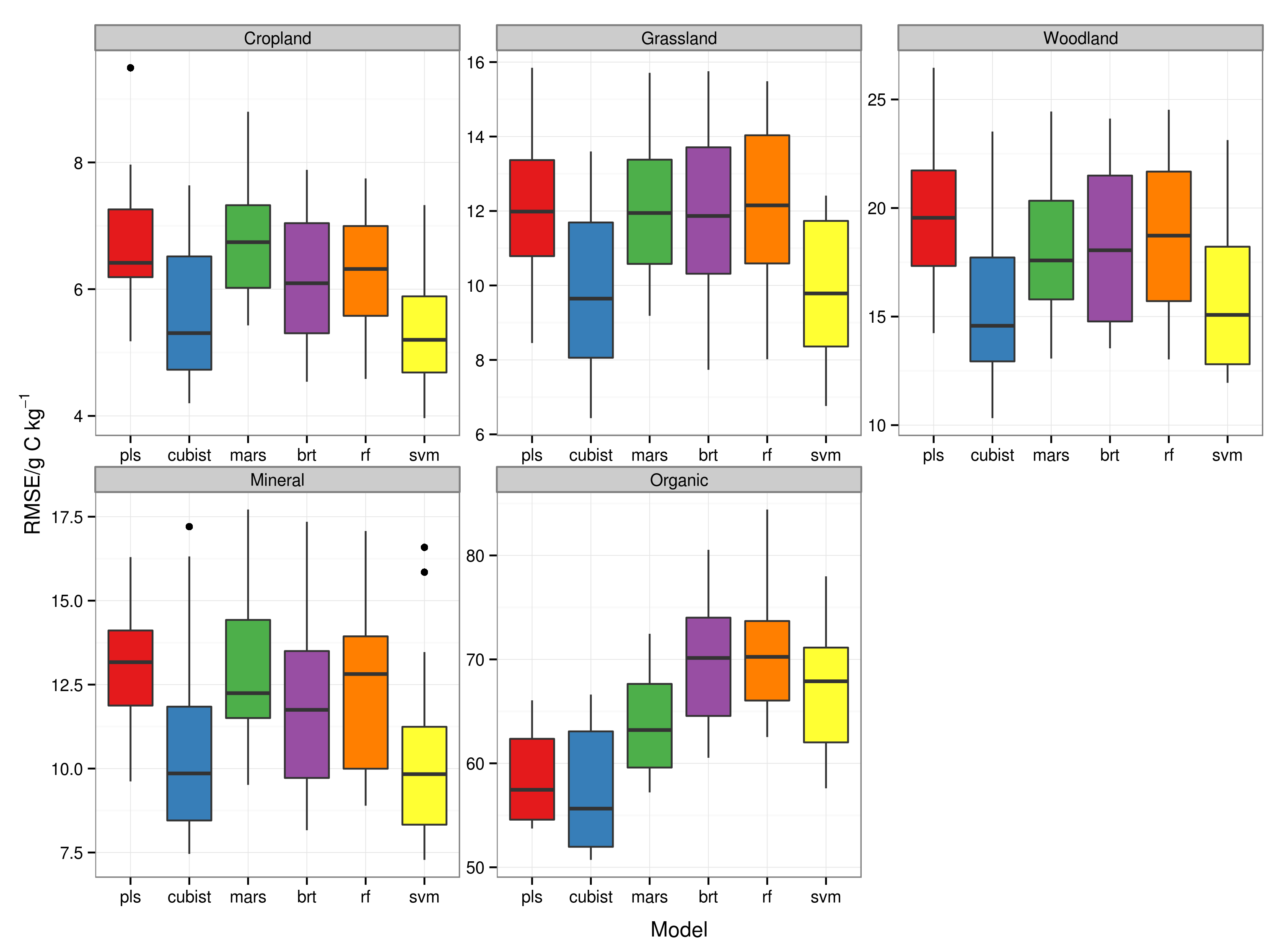

Supplement: Figure S3 — Box-and-whisker plots of the Root Mean Square Error of Prediction ( RMSEP ) as a function of the multivariate calibration approach. Each panel presents separately the results obtained for cropland, grassland, woodland, mineral and organic models. Multivariate models: pls = partial least square regression; cubist = Cubist; mars = multivariate adaptive regression splines; brt = boosted regression tree; rf = random forest; svm = support vector machine. (TIF) [file pone.0066409.s003.tif]
